# A platelet protein biochip rapidly detects an Alzheimer’s disease-specific phenotype

**DOI:** 10.1007/s00401-014-1341-8

**Published:** 2014-09-24

**Authors:** Michael Veitinger, Rudolf Oehler, Ellen Umlauf, Roland Baumgartner, Georg Schmidt, Christopher Gerner, Rita Babeluk, Johannes Attems, Goran Mitulovic, Eduard Rappold, John Lamont, Maria Zellner

**Affiliations:** 1 Center of Physiology and Pharmacology, Institute of Physiology, Medical University of Vienna, Schwarzspanierstrasse 17, 1090 Vienna, Austria; 2 Surgical Research Laboratories, Medical University of Vienna, Vienna, Austria; 3 Department of Medicine I, Institute of Cancer Research, Medical University of Vienna, Vienna, Austria; 4 Institute for Ageing and Health, Newcastle University, Newcastle upon Tyne, UK; 5 Department of Medical and Chemical Laboratory Diagnostics, Medical University of Vienna, Vienna, Austria; 6 Randox Laboratories, Crumlin, Northern Ireland, UK

**Keywords:** Alzheimer’s disease, Diagnosis, Blood platelets, Biomarker, Multiplex protein biochip, Hematologic test

## Abstract

Alzheimer’s disease (AD), a multifactorial neurodegenerative condition caused by genetic and environmental factors, is diagnosed using neuropsychological tests and brain imaging; molecular diagnostics are not routinely applied. Studies have identified AD-specific cerebrospinal fluid (CSF) biomarkers but sample collection requires invasive lumbar puncture. To identify AD-modulated proteins in easily accessible blood platelets, which share biochemical signatures with neurons, we compared platelet lysates from 62 AD, 24 amnestic mild cognitive impairment (aMCI), 13 vascular dementia (VaD), and 12 Parkinson’s disease (PD) patients with those of 112 matched controls by fluorescence two-dimensional differential gel electrophoresis in independent discovery and verification sets. The optimal sum score of four mass spectrometry (MS)-identified proteins yielded a sensitivity of 94 % and a specificity of 89 % (AUC = 0.969, 95 % CI = 0.944–0.994) to differentiate AD patients from healthy controls. To bridge the gap between bench and bedside, we developed a high-throughput multiplex protein biochip with great potential for routine AD screening. For convenience and speed of application, this array combines loading control-assisted protein quantification of monoamine oxidase B and tropomyosin 1 with protein-based genotyping for single nucleotide polymorphisms (SNPs) in the apolipoprotein E and glutathione S-transferase omega 1 genes. Based on minimally invasive blood drawing, this innovative protein biochip enables identification of AD patients with an accuracy of 92 % in a single analytical step in less than 4 h.

**Electronic supplementary material:**

The online version of this article (doi:10.1007/s00401-014-1341-8) contains supplementary material, which is available to authorized users.

## Introduction

Alzheimer’s disease (AD), a multifactorial neurodegenerative disorder, represents the most frequent cause (ca. 60 %) of dementia [8], which has been predicted to impact the quality of life of more than 100 million people by 2050 [1]. Although numerous studies have tried to establish causal links between the pathogenesis of neurodegeneration and dietary, environmental, genetic, and age-related factors [22], the aetiology of AD remains ill-defined. As a result of the heterogeneity of the disease, it has been subdivided into early-onset familial AD (EOFAD) and late-onset AD (LOAD). EOFAD is primarily due to genetic pathology with mutations evident in the genes encoding presenilin 1 and 2 [54] and the amyloid precursor protein (APP) [8]. LOAD, however, is more prevalent (greater than 95 %), has an onset age of at least 65 years, and just one known major genetic risk factor, the *ε4* allele of the *APOE* gene [27]. While genetic testing in combination with familial history helps diagnose EOFAD, valid ante mortem tests for LOAD have yet to be developed. Recently, claims emerged that there is an urgent need to develop objective diagnostic tools that incorporate easily available AD biomarkers [35, 49].

Classic neuropathological characteristics of AD are accumulations of beta-amyloid (Aβ) plaques and neurofibrillary tangles in cortical brain regions [8, 11]. Both features are reflected in cerebrospinal fluid (CSF) since tangles arise as a consequence of increased levels of phospho-tau protein, whereas plaques sequester Aβ peptides and thus lower Aβ concentrations in CSF [29]. Consequently, most studies undertaken to characterize AD-specific biomarkers have focused on these events by analysing CSF. Nevertheless, since the cause of LOAD is multifactorial [36], it is improbable that single (protein) markers can accurately define this complex pathology; an algorithm based on multiple biomarkers should deliver a more accurate clinical diagnosis [35]. Indeed, when applied to AD, it was recognized that a combination of CSF Aβ and (phospho-)tau plus newly discovered candidates offered superior diagnostic accuracy compared to single markers [17]. However, CSF sample collection by lumbar puncture is inconvenient for routine screening. As less invasive alternatives, brain imaging of temporal lobe atrophy, glucose metabolism, or Aβ burden [37] are applied in specialized clinics. However, these methods are expensive and not readily accessible. Therefore, a simple diagnostic screening assay to rapidly and objectively detect AD parameters would be very useful [35]. In particular, a blood test using a minimally invasive sampling route and offering reliable diagnosis by an AD-specific biomarker profile would be a significant clinical advancement, even if detailed clinical patient follow-up would still be required [31, 35]. Whole blood is an attractive sample material since it is a source of cellular and plasmatic proteins that can easily be extracted. Moreover, blood contains platelets, which have increasingly been utilized in the search for AD biomarkers [14, 68]. In fact, platelets are an acknowledged surrogate for neuron physiology since they are the major source of peripheral Aβ [45] and the main storage site of serotonin outside the brain [40]. Furthermore, AD-related changes in APP metabolism [16], monoamine oxidase B (MaoB) enzymatic activity and protein expression have been detected in platelets [4, 75]. Despite a variety of molecular alterations, a comprehensive proteomic analysis of platelets from a large cohort to identify an AD-specific biomarker signature has not yet been performed.

In the present study, we sought to reveal reliable AD biomarkers by two-dimensional differential gel electrophoresis (2D-DIGE) and aimed to develop a high-throughput, routine-applicable analytical system. The latter is fundamentally important in the field of biomarker establishment as it still represents the bottleneck in the translation of research findings into clinical practice [44]. While DNA- and RNA-based microarrays are in widespread use, protein biochips are thus far rarely applied. However, since multiple pathophysiologic events of AD finally take place at the protein level [76], this suggests that phenotyping with a protein biochip might be at least as comprehensive as DNA genotyping or mRNA quantification.

Analysing platelet proteins from AD patients and matched cognitively healthy controls in independent discovery and verification cohorts by 2D-DIGE, we identified five LOAD-regulated protein isoforms which we combined in a sum score. Thus, in this work we report a high-throughput device that has great potential to overcome shortcomings of current AD diagnosis by identification of an AD-specific phenotype in a single analytical step.

## Materials and methods

### Study design and subjects

The cognitive state of 62 clinically suspected LOAD patients was assessed using the neuropsychological test battery of CERAD (Consortium to Establish a Registry for Alzheimer’s Disease) on the day of blood sampling [71]. No patient had been medicated with AD-related therapies such as acetylcholinesterase inhibitors (e.g. donepezil) or NMDA-receptor antagonist (e.g. memantine). Moreover, no patients received antipsychotic drugs or antidepressants. To exclude other causes of cognitive impairment like stroke or tumours, all patients underwent structural brain scanning using MRI, except two patients who were assessed using CT because of claustrophobia or metal implants. Diagnoses were established using the standardized CERAD criteria evaluated from clinical history, brain imaging, and neuropsychological tests [2]. Accordingly, clinical classification of AD patients was defined by two or more deficits in cognition, progressive worsening of memory and other cognitive abilities [3], and onset age between 65 and 85 years. Further selection criteria were severe temporal lobe atrophy on MRI and exclusion of other forms of dementia (i.e. vascular dementia, VaD). In nine patients, clinical diagnosis was neuropathologically confirmed post mortem [48]. Additionally, we included 24 amnestic mild cognitive impairment (aMCI) patients characterized by neuropsychological CERAD testing [21]. Twelve idiopathic Parkinson’s disease (PD) and 13 VaD (four post-mortem-confirmed) patients were also assessed and their cognitive status indicated by mini-mental state examination (MMSE).

MCI patients were selected according to the criteria of the consensus conference in Stockholm in 2003 [69] and the* Diagnostic Manual for Dementia* [3]. Neuropsychological criteria of aMCI were a MMSE greater than 25, not demented, intact activities of daily living, and an impairment in at least two domains of memory with *z* less than −1.0 using diagnostic comprehensive criteria [38]. On the other hand, 112 age- and sex-matched control subjects, who displayed no signs of neurodegenerative and psychiatric diseases, were interviewed, neuropsychologically examined (CERAD), and selected by three experienced psychologists prior to enrolment to exclude cognitive impairment. All individuals were non-smokers. Demographic data and clinical characteristics of the study population are detailed in Tables 1 and OR1 (the latter in the Electronic Supplementary Material).

**Table 1:** Demographic details of AD and control study participants

Demographic variable	Discovery set		Verification set		All
	AD (*n* = 22)	Co (*n* = 25)	AD (*n* = 40)	Co (*n* = 38)	*p* value
Mean age (±SD), (years)	81 (±8.2)	80 (±8.5)	82 (±6.2)	81 (±6.3)	NS (1)
MMSE (SD)	5.5 (±4.2)	29 (±0.8)	14 (±7.1)	29 (±0.9)	<0.001 (1)
% Female	82	86	81	81	NS (1)
% *APOE ε4* ^+/a^	68	8	68	11	<0.001 (2)
% *APOE ε4* ^+/+^	27	0	14	0	<0.001 (2)
Platelet* c* × 10^3^/µl (±SD)	293 (±79)	266 (±58)	220 (±71)	243 (±152)	NS (1)
Education (± SD), (years)	10.4 (±3.0)	10.9 (±2.6)	11 (±3.3)	12.1 (±2.7)	NS (1)

Samples (AD, *n* = 62; Co, *n* = 63) were exclusively derived from non-smokers; subjects with metabolic syndrome and diabetes mellitus type 2 were excluded from analyses. Hypertension was reported for 11 % of AD patients and 19 % of controls; 7 % of AD patients and 5 % of controls were treated with lipid-lowering drugs. Significances of *p* values (1) were calculated with the Mann–Whitney *U* test, significances of genotype distributions (2) by Pearson chi square using ad hoc continuity correction by adding 0.5 to empty cells

*Co* controls, *MMSE* mini-mental state examination, *NS* not significant

^a^Percentage of *APOE ε4*-positivity (homo- or heterozygous)

The study was approved by the ethics commission of the city of Vienna, Austria, EK-04-070-0604 and EK 09/219/1209. Each participant and/or legal guardian was advised of the purpose and procedures of the study and written informed consent was obtained prior to initiating the study in accordance with the principles of the Declaration of Helsinki.

### Neuropathological examination of suspected AD and VaD patients

In nine of the 62 AD patients, clinical diagnosis was neuropathologically confirmed. These patients died 10–18 months after sample collection. Neuropathological diagnoses were made according to established post-mortem consensus criteria for AD, including CERAD scores [52]. AD cases displayed neuropathological changes consistent with Thal phase for Aβ plaques 5.6 [65], CERAD C, and Braak stages V/VI [10], thereby fulfilling the criteria for AD neuropathological changes according to the Alzheimer’s Association guidelines of the National Institute on Aging [53]. As described previously [75], neuropathological examination included haematoxylin/eosin staining, modified Bielschowsky impregnation, as well as tau, Aβ, and α-synuclein immunohistochemistry. VaD in four additional cases was diagnosed following the guidelines by Kalaria and colleagues [41].

### Blood sampling and sample preparation for 2D-DIGE

Blood collection, platelet isolation, platelet protein extraction, total protein concentration determination, and fluorescence labelling for proteome analysis by 2D-DIGE are described elsewhere [67] and detailed in online resource (OR) Information OR1–OR3.

### 2D-DIGE and MS analysis of gel-filtered platelets for biomarker identification

The platelet proteome was investigated by 2D-DIGE in two pH ranges (pH 4–7 and 6–9) on 25.5 × 20.5 cm gels to achieve an optimal protein resolution. 2D-DIGE and image analysis were performed as described previously [70], details are specified in Information OR3. Proteins were identified after tryptic digestion, nanoflow liquid chromatography (1100 Series LC system, Agilent, Palo Alto, CA, USA), and MS/MS fragmentation analysis with an iontrap mass spectrometer (XCT-Plus, Agilent). Details have been published previously [60] and are described in Information OR4.

### *APOE ε4* and GSTO1*A140 genotyping

*APOE ε4* genotyping was performed according to Crook et al. [18], GSTO1*A140 genotyping according to Veitinger et al. [67].

### Sample preparation of PRP for protein biochip

Platelet-rich plasma (PRP) was prepared as described in Information OR1 and subsequently stored at −80 °C. After thawing, 100 µl PRP was centrifuged (3 min, 3,000×*g*) to separate platelets from supernatant platelet-poor plasma (PPP). Ninety microlitres of PPP was mixed with 10 µl of 10× RIPA buffer and incubated (25 min, 4 °C). In parallel, the pelleted platelets were thoroughly resuspended in 20 µl SDS buffer and incubated (25 min, 4 °C). Thereafter, platelet SDS lysates were pooled with 10 µl of RIPA-PPP fraction, with addition of 70 µl 2 % BSA/PBS buffer to bind excess SDS, and incubated (25 min, 4 °C) before application onto the protein biochip. A schematic workflow is presented in Fig. OR1, a protein biochip work instruction overview in Text OR2.

### Statistics

After a Kolmogorov–Smirnov test had confirmed that the data did not show a Gaussian distribution, we selected nonparametric analysis. Mann–Whitney *U* test was used to estimate differences between patients and controls. Sample size determination was based on the algorithm published in our previous study [70]. Statistical significance was set at *p* < 0.05 for all tests and corrected for multiple comparisons [32] of 890 protein spots of the discovery phase (Table OR2) and across both study phases (Table 2). Adjustment was made by the R package “stats”. Only those effects on the platelet proteome that (a) were derived from spots matched in more than 80 % of all 2D gel images, (b) showed an SA ratio (AD/controls) greater than 1.20 or smaller than 0.80, (c) had an unadjusted *p* value less than 0.05 in the discovery phase, and (d) an adjusted *p* value less than 0.05 in the verification phase, as well as (e) across the whole study collective were regarded as significant. Clinical accuracy of examined parameters was assessed using receiver operating characteristic (ROC) curve analysis. ROC blots were constructed and AUC, standard errors, 95 % CI, sensitivity, and specificity calculated. Cut-off values for the best discrimination of positive and negative diagnoses were set by the least squares method using SPSS 20 (SPSS inc, Chicago, USA). Cohen’s *d* was used as a measure of effect size (ES) and calculated with the formula (mean 1 − mean 2)/[(SD 1 + SD 2)/2]. To combine the single AD biomarkers into a score value, sum scores and logistic regressions were calculated using the R package “logistf” for fitting a logistic regression model applying Firth’s correction to the likelihood. Correlations were defined with the Pearson correlation coefficient. Significances of genotype distributions were determined by Pearson chi square using ad hoc continuity correction by adding 0.5 to empty cells.

**Table 2:** AD-related changes in the platelet proteome

Biomarker candidates (*n* = 10)			Discovery		Verification		a.c. AD		All					Biochip
			*n* = 47		*n* = 78		*n* = 9		*n* (AD) = 62; *n* (Co) = 63					
Spot ID (of 890 spots)	UniProt accession#	Protein ID	Ratio (AD/Co)	*p* value (1)	Ratio (AD/Co)	*p* value (2)	Ratio (AD/Co)	*p* value (3)	Ratio (AD/Co)	*p* value (4)	AUC	95 % CI	Effect size (ES)	Final candidates
**B645**	**P27338**	**MaoB**	**1.28**	**<0.001**	**1.46**	**<0.001**	**1.31**	**0.003**	**1.38**	**<0.001**	**0.823**	**0.749–0.898**	**1.27**	^a^
**A1942**	**P02649**	**ApoE3**	**0.57**	**<0.001**	**0.54**	**0.006**	**0.46**	**0.001**	**0.55**	**0.001**	**0.759**	**0.670–0.848**	**0.73**	^a^
**A1855**	**P09493**	**Tm1**	**1.45**	**<0.001**	**1.21**	**0.021**	**1.54**	**0.001**	**1.29**	**0.010**	**0.715**	**0.623–0.806**	**0.76**	^a^
**A1929**	**P02649**	**ApoE4**	**3.31**	**0.001**	**1.88**	**0.021**	**1.96**	**0.020**	**2.22**	**0.001**	**0.797**	**0.694–0.899**	**1.18**	^a^
A921	P00488	Factor XIIIA	1.27	0.003	1.15	0.420	1.30	0.030	1.19	0.296				^b^
B389	P51659	MFE-2	1.46	0.007	1.07	0.889	1.54	0.114	1.21	0.493				^b^
A916	P00488	Factor XIIIA	1.26	0.007	0.87	0.977	1.11	0.681	1.02	0.577				^b^
A2006	P78417	GSTO1*D140	0.72	0.031	0.88	0.246	0.80	0.315	0.80	0.316				^b^
A1663	P60709	Actin, cytoplasmic 1	1.35	0.039	0.83	0.815	1.12	0.662	1.01	0.815				^b^
A2000	P78417	GSTO1*D140	0.70	0.039	0.95	0.831	0.70	0.351	0.84	0.547				^c^
***A1998***	***P78417***	GSTO1*A140												^c^

Protein spots are listed according to their *p* values (1) from the 2D-DIGE discovery phase (AD, *n* = 22; Co, *n* = 25). Four proteins (bold) were also significant in the verification phase (AD, *n* = 40; Co, *n* = 38) after adjusting the *p* values (2) for ten parallel comparisons. Across both study phases (AD, *n* = 62; Co, *n* = 63), the *p* value (4) was adjusted for 890 multiple comparisons. The subgroup of autopsy-confirmed AD (a.c. AD; *n* = 9) was statistically matched with the appropriate controls (same 2D-DIGE gels) and unadjusted *p* values (3) were calculated. Additional AD biomarkers were searched after subdividing the diseased group according to their *APOE ε4* genotype (italic and bold)

^a^Proteins selected for validation with the protein biochip

^b^Candidates that failed verification (*p* value (2))

^c^Biomarker candidates derived by *APOE ε4* stratification. Significances of *p* values were calculated with the Mann–Whitney *U* test

## Results

### Platelet proteome analysis of AD patients

**Fig. 1:**
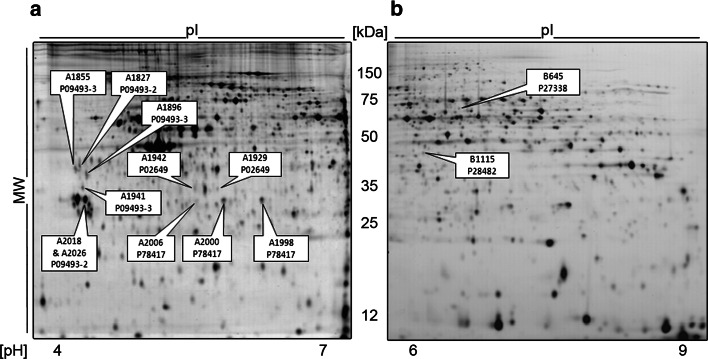
Representative 2D-DIGE array with AD-regulated proteins highlighted: 45 µg total CyDye-labelled platelet protein extracts were separated (15 µg each from an AD patient, a matched control, and the IS) in the pH ranges 4–7 (**a**) and 6–9 (**b**). Spots differentially expressed in AD patients (*n* = 62) and controls (*n* = 63) are marked (spot ID and UniProt number after identification by MS) with ERK2 (spot B1115) as loading control (LC) on the protein biochip

To detect reliable AD-specific biomarkers in platelets, we designed the 2D-DIGE investigation in two stages (Table 1). In the discovery phase, comparison of proteomes derived from 22 AD patients and 25 controls with the applied spot filter criteria revealed ten significantly changed protein spots out of 890 spots matched in more than 80 % of all gels (Table 2). Note that the two inflammation-indicating acute phase proteins C-reactive protein and haptoglobin analysed in plasma of 18 AD patients and 21 controls (discovery cohorts) were not significantly different, indicating no seriously compromised health status due to the disease or biasing comorbidities. To avoid inclusion of false positives (overfitting), verification of the ten identified candidates was sought using 40 newly recruited AD patients and 38 controls. Consequently, the filter criteria in this study phase were set to adjusted *p* values less than 0.05 for ten comparisons. At this stage, the top four ranked protein spots were confirmed by unadjusted and adjusted *p* values [32]. Likewise, these AD-related isoforms had an adjusted *p* value less than 0.05 if corrected for 890 comparisons when calculated across the whole study cohort (Table 2). Six biomarker candidates from the discovery phase could not be verified and were excluded from further analyses. The most significant (*p* = 3.42 × 10^−7^) expressional upregulation in AD patients was that of MaoB spot B645 (Figs. 1, OR2b). ApoE spot A1942 demonstrated a decreased expression and was attributed to the ε3 isoform (Figs. 1, OR2a) (*p* = 0.0009). Accordingly, this spot exhibited lower SA in *ε4*-positive patients. The increased ApoE spot A1929 was assigned to the ε4 isoform (*p* = 0.001). The fourth strongest confirmed AD-regulated spot A1855 was identified as tropomyosin 1 (Tm1) (*p* = 0.008). Adjacent spots A1827, A1896, and A1941 (Fig. 1) were also recognized as Tm1 (Fig. OR2c). Glutathione S-transferase omega 1 (GSTO1) spots A2000 and 2006 (Fig. 1) exhibited reduced expressions; however, this was not confirmed in the verification phase (Table 2). Nonetheless, there was a strong association with the *APOE* genotype in AD patients which we assessed in more detail.

### Non-*APOE ε4* AD patients overrepresent GSTO1 isoform A140

Since studies have reported distinct biochemical profiles of AD sufferers with respect to their *APOE* genotype [61], we genotyped all subjects and subdivided them into *APOE ε4* carriers and non-carriers. As expected, the AD group included significantly more *APOE ε4* carriers (68 %) than the control group (11 %). All confirmed AD-related protein spots were also significantly changed in these subgroups (Table OR2). The two GSTO1 spots A1998 and A2000, previously identified by our laboratory as isoforms of SNP rs4925 [67], displayed significant modulation in *APOE ε4* non-carriers (Table OR2): spot A1998 was upregulated to 1.61 (*p* = 0.020) in *ε4*-negative patients, isoform A2000 was significantly decreased to 0.41 (*p* = 0.037). The lower abundant isoform A2006 was not significantly downregulated to 0.43 (*p* = 0.095). Similarly, all three autopsy-confirmed (a.c.) *APOE* *ε4*-negative patients expressed exclusively GSTO1*A140. Furthermore, neuropathology assigned three *APOE* *ε4*-negative probable AD patients, all of which were heterozygous for SNP rs4925, as exhibiting vascular dementia. Additionally, the top four proteins displayed highly significant expression changes also in the a.c. AD subgroup (Table OR2). Consequently, all these markers were included in the AD panel together with the GSTO1 isoforms.

### Validation of GSTO1*A140D distribution by PCR analysis of SNP rs4925

To underscore the above finding, we genotyped all participants and confirmed the 2D-DIGE data (Fig. OR3) that exclusively two GSTO1*A140 alleles were present in non-*APOE ε4* AD patients (*n* = 20) as compared to 32 % in *APOE ε4*-positive patients and 38 % in controls (30 % in non-*APOE ε4* controls).

### Models of AD biomarker combinations

In order to establish the most powerful biomarker algorithm to identify AD samples, we reviewed different combinations of the significantly changed proteins/isoforms (Table 3). Combinations for optimal distinction between diseased and healthy were calculated separately for the discovery and verification sets, and for the whole collective using primary sum scores. For these scores, we integrated the *APOE* *ε4* allele count instead of SA since there was considerable background noise in the area of the 2D-DIGE ApoE4 spot A1929 (Fig. 1) in *ε4*-negative individuals. The sum score of the top-ranked protein MaoB and the *APOE ε4* allele count (model 2) increased the AUC of MaoB alone (model 1) from 0.823 (ES = 1.27) to 0.896 (ES = 1.80). Inclusion of Tm1 A1855 (model 3) moderately improved this AUC to 0.904 (ES = 1.93). Addition of GSTO1*A140 SA (model 4), overrepresented in *APOE ε4* non-carriers, lowered the AUC to 0.901 (ES = 1.81). Most importantly, introducing the *APOE ε4* allele into a decision tree (Table 3, model 5) yielded the highest AUC of 0.969 (95 % CI = 0.944–0.994, ES = 2.50). With this model, we could differentiate patients from controls with 94 % sensitivity and 89 % specificity. Consequently, this study utilized two algorithms dependent on the absence (5a, addition of GSTO1*A140) or presence of at least one *APOE* *ε4* allele (5b, addition of GSTO1*D140). This model demonstrated a robust performance in the discovery (AUC = 0.952) and verification (AUC = 0.980) phase with a high separation power independent from gender. Similarly, the combination of these biomarkers by logistic regression yielded an AUC of 0.966 (95 % CI = 0.940–0.991, cut-off = 0.510: sensitivity = 92 %, specificity = 86 %).

**Table 3:** Performance of different biomarker combinations of discovery, verification, and pooled sample sets

Biomarker algorithm							Statistics				
Model	Algorithm	Standardised abundances of 2D-DIGE				Allele count	Discovery (*n* = 47)	Verification (*n* = 78)	All (*n* = 125)		
		MaoB B645	Tm1 A1855	GSTO1*A140 A1998	GSTO1*D140 A2000	*APOE ε4*	AUC	AUC	AUC	95 % CI	ES
0	0	−	−	−	−	+	0.797	0.782	0.787	0.704–0.869	1.40
1	1	+	−	−	−	−	0.838	0.821	0.823	0.748–0.898	1.27
2	2	+	−	−	−	+	0.865	0.912	0.896	0.842–0.955	1.80
3	3	+	+	−	−	+	0.890	0.910	0.904	0.851–0.956	1.93
4	4	+	+	+	−	+	0.893	0.916	0.901	0.849–0.954	1.81
**5**	**5a**	**+**	**+**	**+**	−	−	**0.952**	**0.980**	**0.969**	**0.944–0.994**	**2.50**
	**5b**	**+**	**+**	−	**+**	**+**					
**6**	**6a**	**+**	**+**	**+** (*0.6)	−	−	**0.944**	**0.949**	**0.947**	**0.884–0.998**	**2.36**
	**6b**	**+**	**+**	−	**+** (*0.9)	**+**					

Spot SA (2D-DIGE) of the significant platelet proteins MaoB and Tm1 (A1855) were combined with the *APOE ε4* allele count by summation. In the split algorithms (a and b), GSTO1*A140 SA was added to *APOE ε4*-negative samples, GSTO*D140 SA to *APOE ε4*-positive samples. ROC curves were calculated for the discovery (*n* = 47), verification (*n* = 78), and pooled (*n* = 125) sample sets. Biomarker combinations marked in bold were the best for 2D-DIGE (model 5) or the protein biochip (model 6) with highest AUCs. Model 6 simulates the design of the developed protein biochip, whereby instead of GSTO1 SA the allele counts (adjusted with a coefficient according to their 2D-DIGE abundance) were taken

### Accuracy of model 5 algorithm for identification of AD patient

To investigate whether AD patients can be discriminated from other neurodegenerative disease patients, we analysed 24 aMCI, 12 PD, and 13 VaD patients (Table OR1) by 2D-DIGE. Using the model 5 algorithm, AD patients could be separated from PD with high (AUC = 0.912) and from VaD with still moderate (AUC = 0.738) precision. Remarkably, differentiation of post-mortem-confirmed VaD cases (*n* = 4) from AD subjects was even more pronounced (AUC = 0.915). An AUC of 0.798 could be achieved for discrimination of aMCI patients and controls (Table OR3).

### Development of a novel protein biochip for AD detection

**Fig. 2:**
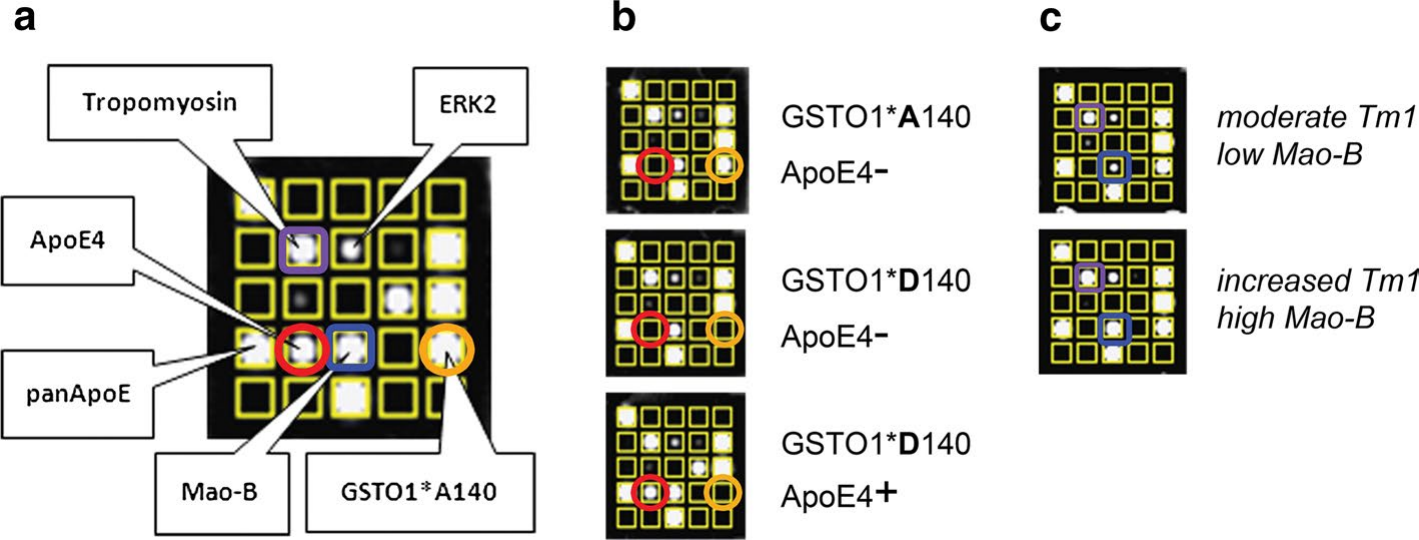
Schematic representation of the new AD multiplex protein biochip. **a** Antibodies directed against the proteins of interest were spotted on the biochip, incubated with samples (or calibrators) and target analyte concentrations quantified by measuring chemiluminescence signals of bound HRP-labelled secondary antibodies. **b** Quantification of GSTO1*A140 (*orange circles*) and ApoE4 (*red circles*) with the protein biochip. Together with the image in **a**, all four possible genotypes (*APOE ε4* ^−^/GSTO1*A140, *APOE ε4* ^+^/GSTO1*A140, *APOE ε4* ^−^/GSTO1*D140, *APOE ε4* ^+^/GSTO1*D140) are shown. **c** Quantitative protein expression differences of Tm1 (*purple squares*) and MaoB (*blue squares*)

To enable high-throughput analysis of the identified specific AD phenotype, we engineered a protein biochip combining protein quantification and proteomic genotyping (Fig. 2). This included establishment of a new sample preparation method (Fig. OR1) for simultaneous quantification of plasmatic, cytosolic, and membrane proteins, implementation of an additional assay for a stably expressed loading control (LC) in order to optimize sample normalization, and development of highly specific peptide antibodies to discriminate protein isoforms. We raised monoclonal antibodies against AD-related proteins in-house and confirmed high specificity and functionality on 2D-WB membranes (Figs. OR2a, OR2b; Text OR1).

Low biological variation proteins were systematically evaluated as potential LCs in psychiatrically diseased and healthy subjects [6] to compensate for variation in platelet numbers which was observed to be 36 % in 102 PRP samples (363 ± 131 × 10^3^ platelets/µl). LCs circumvent inconvenient platelet counting and total protein determination which is not possible because of high plasma protein content in PRP lysates (Fig. OR1). ERK2 was selected as LC for one-step normalization on this biochip because of its AD-independent expression (Table OR2; Fig. OR4), its smooth technical performance on the biochip, and its previous use as LC on WB for Aβ-activated microglia [62]. Biochip feasibility studies of ERK2-normalization were performed by repeated analysis of different dilutions of highly concentrated endogenous PRP samples (Fig. OR5). Linear regression analysis of ERK2 concentration against the respective platelet number demonstrated a high correlation coefficient (*r* = 0.99).

The advantage but also challenge of a multiplex array is the combination of several assays on a single platform. A detailed description of the protein biochip technology has been published previously [23], the assembly of the novel AD biochip is outlined in Fig. 2a. One pair of antibodies was required for each target protein, comparable to a sandwich ELISA. Calibration curves with affinity purified proteins for each individual assay are presented in Fig. OR6.

An easy sample preparation protocol applicable for routine analysis was developed and is detailed in Fig. OR1 and Text OR2. Notably, a simple SDS buffer was superior to several other commonly used detergents, compatible with all assays, and most efficient in extracting the membrane protein MaoB. To quantify proteins released by platelets during freezing/thawing and to facilitate protein-based *APOE* genotyping (higher abundance in plasma), a plasmatic fraction was included. Separate treatment of platelets and PPP with subsequent fusion enables parallel analysis of cellular, membrane, and plasmatic proteins and permits introduction of a dilution factor for much higher abundant plasma proteins.

All available 102 samples previously analysed by 2D-DIGE were measured with the protein biochip: 21 pairs of the discovery set and 30 pairs of the verification set. Using the protein biochip for determination of GSTO1*A140 and *APOE* *ε4* allele counts (Fig. 2a, b), 98 % of all samples (100 out of 102) were correctly genotyped for *GSTO1* SNP rs4925 and 100 % correct genotyping was achieved for *APOE ε4* by normalization with either ERK2 or panApoE concentrations. *APOE ε4* stratification confirmed the high prevalence of GSTO1*A140 as all 16 *APOE ε4*-negative AD patients exhibited the GSTO1*A140A genotype (vs. 25 % of *APOE ε4*-negative controls). Moreover, biochip analysis replicated the higher expression of both quantitative markers Tm1 and MaoB (Fig. 2c) in patients as compared to controls. The 18 % increase of Tm1 was already significant without normalization (*p* = 0.003), the 13 % elevated MaoB level was not (*p* = 0.121). After correction with ERK2, both *p* values significantly improved (*p*_Tm1_ = 0.001; *p*_MaoB_ = 0.006), as well as the MaoB upregulation to 17 %.

To establish a biochip sum score (Table 3, model 6), we also divided ERK2-normalized MaoB and Tm1 concentrations by their respective average concentrations in order to obtain relative values like in 2D-DIGE before addition to the allele counts. Since the array was designed to genotype samples for GSTO1*A140 and *APOE ε4* rather than absolutely quantifying the protein abundances, the respective allele numbers were used in model 6. According to their 2D-DIGE SA, weighting factors of 0.6 and 0.9 for isoforms *A140* and *D140* were introduced. The ES of 2.36 was comparably strong as that of 2D-DIGE model 5 and the AUC of 0.947 (95 % CI = 0.884–0.998) was just slightly lower.

**Fig. 3:**
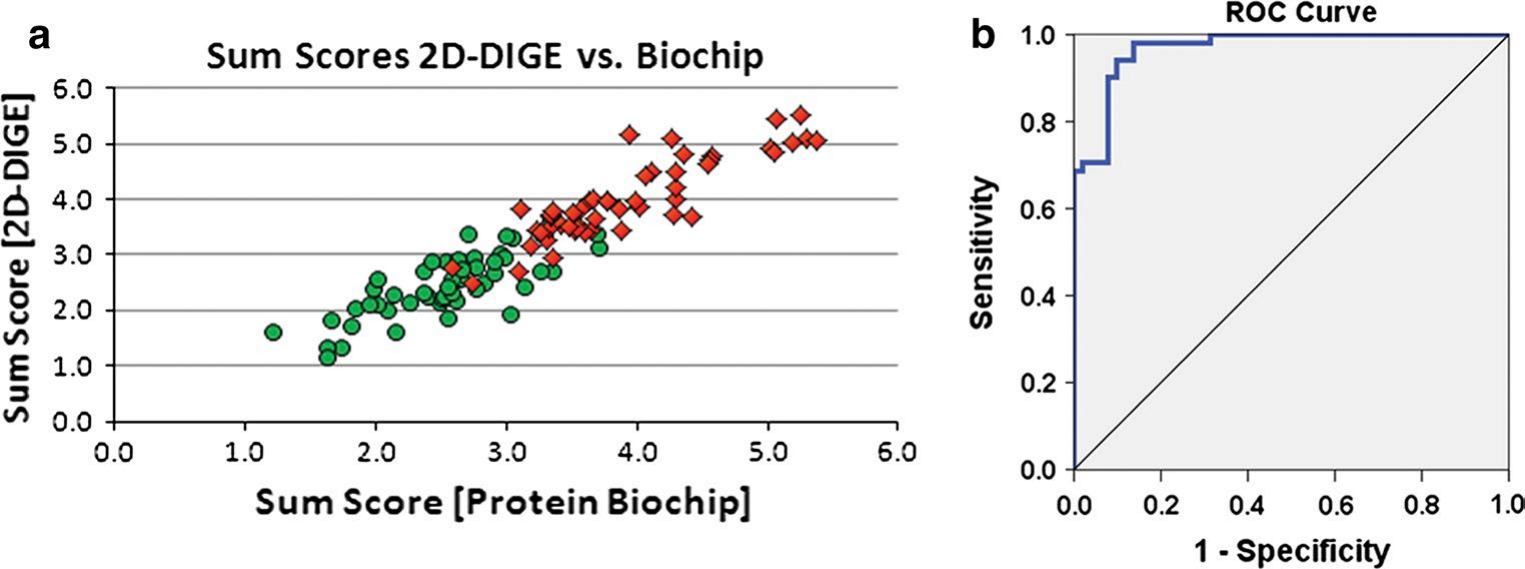
Statistical analysis of 51 AD and 51 control samples with 2D-DIGE and the protein biochip. **a** Scatter plot of sum scores (arbitrary units, *n* = 102) derived by addition of *APOE ε4* and *GSTO1* allele counts to MaoB and Tm1 concentrations (models 5 and 6 of Table 3). Protein biochip sum scores are plotted on the *x* axis, those of 2D-DIGE on the *y* axis. *Red squares* AD samples; *green circles* control samples. **b** ROC curve of the logistic regression calculated for the 102 clinical samples analysed with the protein biochip (AUC = 0.969)

The separation of patients and controls, as well as the correlation of the protein biochip with 2D-DIGE is presented in Fig. 3a. However, since a sum score is not practical for routine biochip application, we additionally calculated a logistic regression model which yielded an AUC of 0.969 (95 % CI = 0.941–0.996; sensitivity = 94 %, specificity = 90 %, Fig. 3b). In summary, we established a novel high-throughput platform that achieved AD diagnosis with an accuracy of 92 %.

## Discussion

Systematic characterization of the platelet proteome by 2D-DIGE identified a reliable AD blood biomarker signature which we translated into a protein biochip array with great feasibility for routine diagnosis. In this study, we revealed GSTO1 as a novel AD biomarker since the A140 isoform was significantly overrepresented in *APOE ε4*-negative AD patients. Accordingly, the variant of SNP rs4925, D140 [5, 12, 47, 55], was underrepresented in this AD subgroup. Further, we identified significant protein expression changes apparently not linked to genetic mutations: upregulated Tm1 isoforms represent novel peripheral diagnostic targets. Previous studies have demonstrated that tropomyosin is an integral part of neurofibrillary tangles [26] and increased expression has been detected in the olfactory bulb of aged mice [57]. In humans, olfactory impairment is associated with normal aging and several age-associated neurodegenerative disorders, including AD and PD [58]. Elevated levels of Tm1 have been quantified in periventricular white matter [13] and the glycosylated hippocampal proteome [20]. Higher concentrations of oxidatively modified tropomyosin isoforms have been found in the choroid plexus of AD patients [56]. Oxidative modification of Tm by reactive oxygen species (ROS) produced by monoamine oxidase has been linked to myofibre damage in muscular dystrophy [50]. Mechanistically, formation of disulphide cross-bridges reduces protein solubility and may enforce the generation of neurofibrillary tangles in AD. In parallel, studies have chronicled increased activities and protein concentrations of the dopamine-degrading enzyme MaoB in AD; the latter could repeatedly be confirmed in the present study (Table 2). The described generation of ROS in turn might boost the amyloidogenic pathway e.g. via increasing the activity of BACE1 in AD cases [9]. In contrast to ApoE4 and GSTO1*A140, detailed examination failed to established any correlation of MaoB expression with its most frequently described SNP rs1799836 [39]. Instead, MaoB concentrations correlated with smoking [42], ageing [75], and inversely with plasma vitamin B_12_ concentration [74]. Therefore, MaoB expression might indicate a functional molecular link of lifestyle and AD pathogenesis potentially via epigenetic regulation through the one-carbon metabolism [74]. A mechanistic hypothesis on the elevated expression of MaoB [42], Tm1 [72], and BACE1 [25] is a deregulated one-carbon metabolism which leads to reduced promoter methylation and, consequently, to increased protein expression. Regarding the tau pathology, a reduced methylation of protein phosphatase 2A with concomitant tau hyperphosphorylation has been described [63].

The highly significantly altered expression of ApoE4 in the AD platelet proteome confirmed this SNP as the most powerful risk factor for LOAD besides ageing. However, the relatively low AUC of 0.787 (Table 3, model 0) for the *APOE ε4* allele count reflects limited diagnostic sensitivity/specificity [7]. In accordance with previous studies, we found that 10–16 % of cognitively healthy elderly carry at least one *ε4* copy [59], while it is present in about 60 % of autopsy-confirmed AD patients [43]. A similar *APOE ε4* distribution has also been published in the ADNI study [15]. However, this indicates that roughly half of all AD patients are *APOE ε4*-negative and alternative biomarkers specific for this subgroup are required. *APOE ε4* stratification revealed significant changes in the distribution of the two GSTO1 protein spots A1998 and A2000 in *APOE ε4*-positive and *APOE ε4*-negative AD patients (Fig. OR3; Table OR2). Initially, GSTO1*D140 was reported to be associated with a later age-at-onset [46]; however, follow-up studies could not confirm this finding [5, 12, 55]. Notably, none of these studies presented data about the SNP rs4925 distribution in *APOE ε4*-negative AD samples. GSTO1 has diverse functions, including mitigation of oxidative stress, and may underlie the pathophysiology of several neurodegenerative diseases. Recently, it has been shown that GSTO1*D140 has a higher glutathionylation activity than GSTO1*A140, thereby potentially preventing oxidative damage of proteins [51]. A protective effect of the *D140* allele has been reported for PD [5, 47].

Here, we found platelet MaoB to be the most powerful biomarker in the differentiation of healthy and diseased (ES = 1.30), corroborating previously published data [74]. MaoB had a higher ES than abnormally processed platelet APP in moderate AD patients (ES = 1.10) [73]. The diagnostic performance of Tm1 A1855 (ES = 0.76) was comparable with decreased BACE1 levels (ES = 0.85) [19], decreased platelet phospho-GSK3B levels (ES = 0.68) [24], or plasma Aβ42/Aβ40 ratio (ES = 0.80) [30]. Although all of these AD biomarkers differed significantly between AD and controls, none of them reached the sensitivity and specificity claimed [35, 66]. Several studies focusing on combinations of biomarkers revealed higher discriminating power for algorithms than for single candidates [17, 34, 77]. Likewise, unifying the well-known AD biomarkers MaoB and *APOE ε4* with the novel candidates Tm1 and GSTO1 (Table 3) generated a highly disease-specific test (AUC = 0.969). This diagnostic accuracy of 92 % better conforms with clinical requirements for dementia diagnosis [66] and has a similarly good diagnostic performance as established CSF biomarkers [64]. The scores in Table 3 furthermore indicate similar diagnostic accuracy independent of the stage of AD patients: AUC values were comparably high for late stage (mean MMSE discovery set = 5.5 ± 4.2) and mild/moderate stage (mean MMSE verification set = 14 ± 7.1) AD patients as evident in e.g. model 5 with AUC values of 0.952 (discovery phase) and 0.980 (verification phase). Additionally, first comparisons with subjects suffering from PD, VaD, or aMCI indicate that this algorithm is fairly specific for AD and may already indicate prodromal disease stages. Consequently, we engineered and validated a multiplex system for high-throughput analysis. Several technical issues had to be considered: an optimal lysis procedure is defined by the biochemistry of the proteins of interest and the analytical platform (Fig. OR1). While mild buffers are appropriate for solubilisation of cytosolic proteins and determination of enzymatic activity, stronger detergents are required to extract membrane proteins. The use of SDS for cell lysis is well established and has already been applied to blood platelets [33]. However, reports about a sole SDS-based lysis of pelleted platelets are sparse as SDS treatment is almost exclusively used for subsequent matrix-based protein separation techniques such as SDS-PAGE. Detection of soluble SDS-extracted proteins by ELISA is an exception [28]. Nevertheless, ionic SDS was the most effective detergent and rendered compatible with all assays on the protein biochip. Integration of an LC has ensured that this is the first device to offer multiplexed quantification of cellular and plasmatic proteins in a single analytical step. These technical innovations are not limited to AD diagnosis but have a wide field of further applications.

Translation of our 2D-DIGE data to the new protein biochip yielded analogous results: analysis of a well-defined AD collective versus healthy controls generated high accuracy of 92 % (Fig. 3). With this carefully validated diagnostic kit, sample sizes including that of MCI and other dementia subtypes, to determine the broader efficacy of this platelet array, can be increased in the future. Of particular interest is further assessment of the pathologic significance of this platelet biomarker panel in patients with incipient AD and follow-up of aMCI patients. In summary, we demonstrate the utility of measuring multiple analytes from a PRP preparation in a single step to aid the diagnosis of LOAD.

## Electronic supplementary material

Below is the link to the electronic supplementary material.

Supplementary material 1 (PDF 870 kb)
