# Supplementary material for: A platelet protein biochip rapidly detects an Alzheimer’s disease-specific phenotype
Source: Acta Neuropathol. 2014 Sep 24;128(5):665–77. doi: 10.1007/s00401-014-1341-8 (PMC4201753; doi:10.1007/s00401-014-1341-8)
Supplement: Supplementary file 1 — Supplementary material 1 (PDF 870 kb) [file 401_2014_1341_MOESM1_ESM.pdf]

A platelet protein biochip rapidly detects an Alzheimer's disease-specific phenotype

Acta Neuropathologica

Authors:

Michael Veitinger, Rudolf Oehler, Ellen Umlauf, Roland Baumgartner, Georg Schmidt, Christopher Gerner, Rita Babeluk, Johannes Attems, Goran Mitulovic, Eduard Rappold, John Lamont, Maria Zellner

*Corresponding author:

Maria Zellner, Institute of Physiology, Center of Physiology and Pharmacology, Medical University of Vienna, Schwarzspanierstrasse 17, 1090 Vienna, Austria; E-mail: maria.zellner@meduniwien.ac.at

Online Resource (OR)

Information OR1 Blood sampling and isolation of platelets

Antecubital vein blood, drawn without stasis, was collected in vacuum tubes (Greiner Bio-One, Kremsmuenster, Austria) coated with 0.129 mol/L sodium citrate. To avoid venipuncture tissue contamination, the first tube was discarded. A MicroDiff 18 blood analyser (Coulter Electronics, Miami, USA) was used to determine platelet counts of platelet-rich plasma (PRP) and gel-filtered platelets (GFP). PRP was generated by a soft centrifugation step (20 min, 120x*g*, room temperature, without brake). For 2D-DIGE analysis, GFP were prepared from PRP and for protein biochip analysis, PRP was frozen at -80 °C. GFP were isolated by size exclusion chromatography with Sepharose 2B (GE Healthcare) as described previously [5].

Information OR2 Preparation of gel-filtered platelets for 2D-DIGE

Platelet protein were extracted from gel-filtered platelet (GFP) suspension by trichloroacetic acid and subsequently washed four times with ice-cold acetone [8]. Pellets were resolubilised by shaking (18 h, 4 °C) in 70 µl denaturing 2D sample buffer (7 M urea, 2 M thiourea, 4% CHAPS, 20 mM Tris-HCl, pH 8.5) per 100 x 10^6 platelets. Protein concentrations were determined in triplicate using a Coomassie brilliant blue protein assay kit (Pierce Biotechnology, Rockford, IL, USA). All samples were diluted 1/20 with PBS to avoid interference of the 2D sample buffer with the protein assay. Accordingly, 2D sample buffer was added to BSA standards at a final 5% concentration. The internal standard (IS) was a platelet protein pool of all study participants. IS and samples were aliquoted and stored at -80 °C. Proteins were labelled prior to electrophoresis with fluorescent cyanine dyes (CyDyes, GE Healthcare, Uppsala, Sweden): per µg of protein, the IS was labelled with 5 pmol Cy2, samples were labelled with 5 pmol Cy3 or Cy5.

Information OR3 2D-DIGE analysis of GFP for biomarker identification

For alkaline proteins, Immobiline IPG-DryStrips (24 cm, pH 6-9, GE Healthcare) were rehydrated in 450 µl modified rehydration solution (7 M urea, 2 M thiourea, 150 mM DTT, 2% ampholytes pH 6-9) [7]. Isoelectric focusing (IEF) using an IPGphor unit (GE Healthcare) was completed after cup-loading of 15 µg Cy2-, Cy3-, and, C5-labeled protein samples each and reaching 30 kVh. Resolution of acidic proteins (24 cm, pH 4-7, GE Healthcare) was accomplished passively with in-gel rehydration of 45 µg (three samples, 15 µg each) CyDye-labelled protein extracts in a similar rehydration buffer substituted with 70 mM DTT and 0.5% ampholytes, pH 4-7 (Serva, Heidelberg, Germany). IEF was performed as described above. Proteins were separated in the second dimension using 11.5% SDS-PAGE (35 V for 1 h, 50 V for 1.5 h, and finally 110 V for 16.5 h at 10 °C) and images with a resolution of 100 µm were acquired on a Typhoon 9410 imager (GE-Healthcare, Freiburg, Germany). The DeCyder software module 'Differential In-gel Analysis' (version 6.00.28; GE Healthcare) was applied to detect spots on the images with a target spot number of 2500. All sample gels were matched with the master gel in the DeCyder module 'Biological Variation Analysis' (version 6.01.02). Detailed gel image analysis has been described previously [6]. The SA of a protein spot in each sample was calculated relative to the same spot of the IS to reduce inter-gel variation.

Information OR4 Protein identification by mass spectrometry (MS)

Protein identification by MS was completed after preparation of tryptic protein hydrolysates as described previously [4]. Briefly, peptides were loaded on a Zorbax 300SB-C8 (5 µm, 0.3 mm x 5 mm) column and separated by nanoflow liquid chromatography (1100 Series LC system, Agilent, Palo Alto, CA, USA) with a Zorbax 300SB-C18 (5 µm, 75 µm x 150 mm) column. The flow-rate of a gradient from 0.2% formic acid and 3% acetonitrile to 0.2% formic acid and 45% acetonitrile over 12 min was set to 250 nl/min. Spectrum Mill MS Proteomics Workbench software (Version A.03.03, Agilent) was used to interpret MS/MS data UniProt database for human proteins (version 14.3, containing 20,328 entries) was searched, allowing for a precursor mass deviation of 1.5 Da, a product mass tolerance of 0.7 Da and a minimum matched peak intensity (%SPI) of 70% and one missed cleavage. Cysteine carbamidomethylation was set as fixed modification because of previous chemical modification. The false discovery rate for peptides scoring higher than 13 was consistently less than 1%, generating a certainty better than 99.9% for those proteins identified with two or more peptides.

Figures

Fig. OR1 Schematic sample preparation workflow

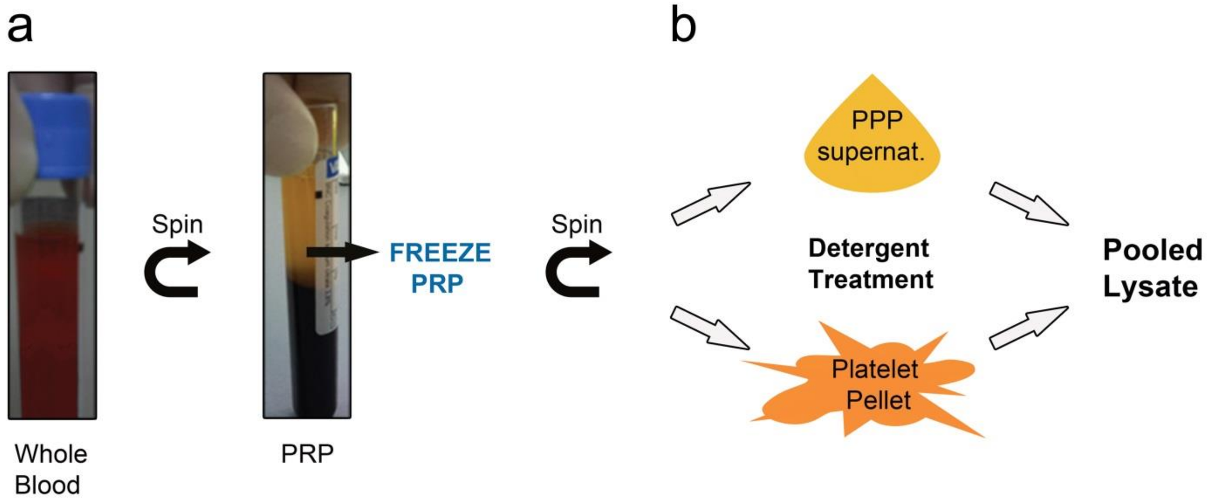

(a) After collection of whole blood in clinics, only a single centrifugation step is required to separate platelet-rich plasma (PRP) from the haematocrit. The supernatant PRP can be stored at -80 °C. **(b)** For concomitant detection of cellular and plasmatic proteins in the analytical laboratory, pelleted platelets were treated with a SDS-lysis buffer, whereas RIPA lysis buffer was added to supernatant platelet-poor plasma (PPP) for lysis of remaining platelets and/or shed microparticles. The two fractions were pooled in the desired ratio and applied to the protein biochip.

Fig. OR2 Validation of antibodies directed against AD biomarker candidates by 2D-WB

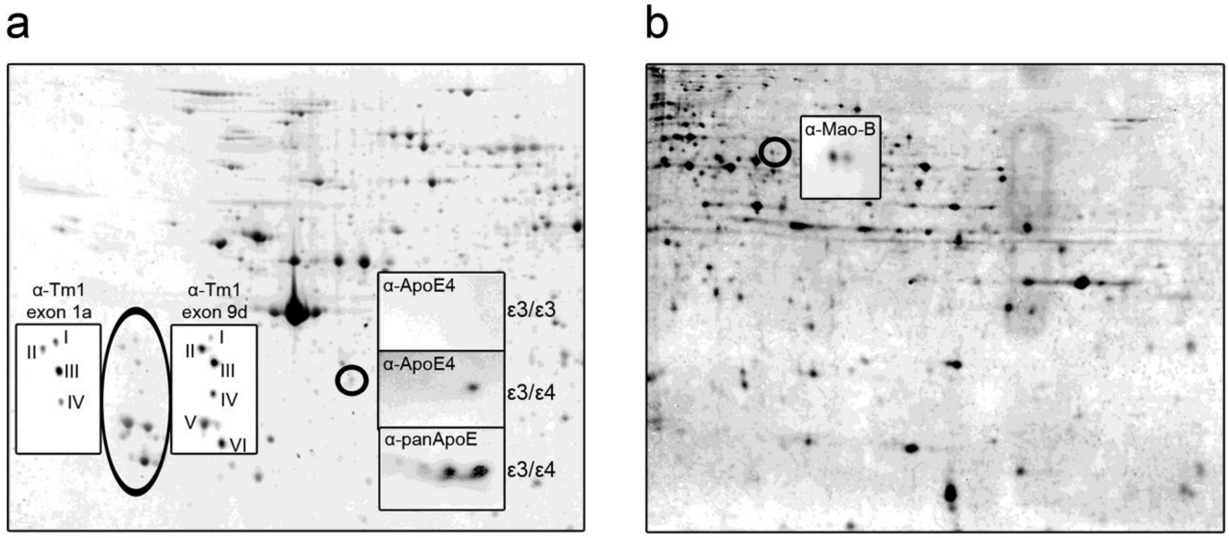

c

Spot ID	Accession No.	Protein ID	Ratio AD/Co	*p*
I = A1827	P09493	Tropomyosin 1	1.12	0.009
II = A1855	P09493	Tropomyosin 1	1.29	< 0.001
III = A1896	P09493	Tropomyosin 1	1.07	0.080
IV = A1941	P09493	Tropomyosin 1	1.04	0.233
SUM	P09493	Tropomyosin 1	1.13	0.018

Representative 2D-images of the platelet proteome (**(a)** pH 4-7, **(b)** pH 6-9) blotted onto nitrocellulose membranes and visualized by total protein stain [7]. Spots of interest are labelled with circles, the squared cut outs are details of 2D-WB membranes probed with the particular antibodies. The 2D-images of specific tropomyosin antibody signals were overlaid to the respective total protein stain of the blotted proteins to validate MS identifications. ApoE spots were not visible in the total protein stain but the position is marked since a further protein spot is located at the same site. Tm1 spots: I=A1827, II=A1855, III=A1896, IV=A1941, V=A1987 (P67936; Tropomyosin alpha-4 chain, unchanged in AD), VI=A2018 (P09493-2). **(c)** summarises the different Tm1 isoforms.

Fig. OR3 Distribution of GSTO1*A140 in controls, *APOE ε4*-negative and -positive AD patients

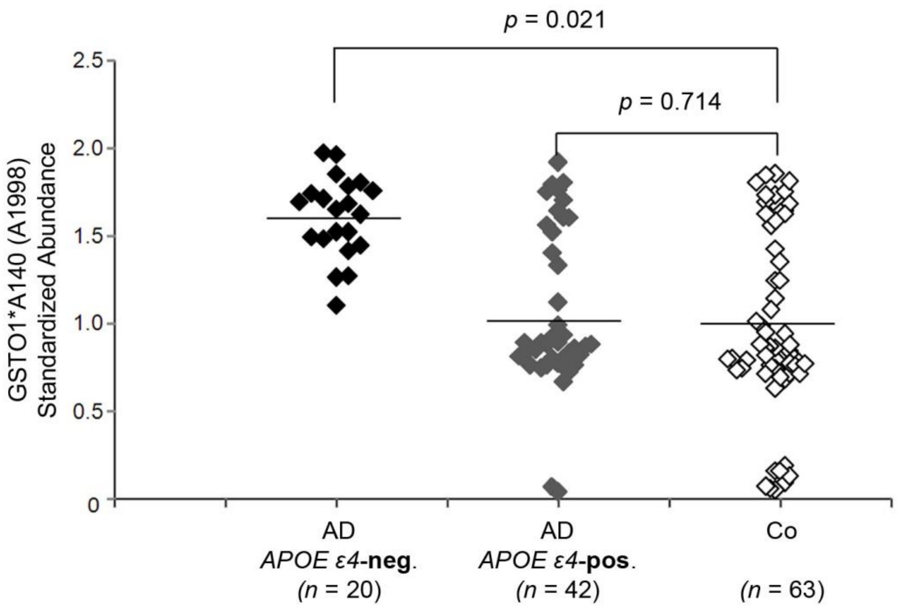

AD patients (*n*=62) were sub-grouped according to their *APOE ε4* genotype and compared against age- and sex-matched cognitively healthy controls (*n*=63). All *APOE ε4*-negative patients (*n*=20) displayed similar GSTO1*A140 SA, two different expression levels for homo- and heterozygous samples were detected in *APOE ε4*-positive patients (*n*=42) and controls (*n*=63). Significant genotype correlation was evident with the SA of GSTO1*A140 (*r*=0.88) and *D140 (*r*=0.92) protein isoforms. *p*-values were calculated with the Mann-Whitney *U* test and adjusted for the number of multiple comparisons of 890 matched protein spots.

Fig. OR4 One-dimensional WB of platelet protein extracts obtained from AD patients and controls

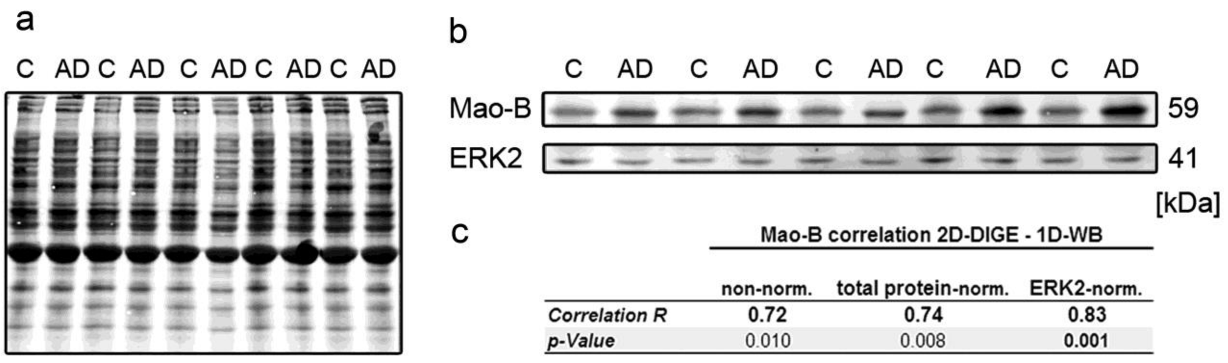

ERK2 is an easily accessible and well characterized cytosolic protein that exhibits low variation in AD and control platelets [1;2]. **(a)** Total protein stain (15 µg/lane) of five AD and five control (C) platelet samples. (**b**) The membrane was probed with specific antibodies to visualize ERK2 (41 kDa) and MaoB (59 kDa). **(C)** Protein bands were quantified with Image J. MaoB signals were correlated with the 2D-DIGE SA of respective samples either without normalization or after normalization with total protein concentration or ERK2 concentration.

ERK2 concentrations in platelets from AD patients and controls were identical, just as the total protein concentrations. MaoB normalization with ERK2 improved the correlation between 2D-DIGE and 2D-WB (*r*=0.83, *p*=0.001), relative to non-normalized signals (*r*=0.72, *p*=0.01).

Fig. OR5 Endogenous ERK2 concentrations of PRP dilutions measured with the protein biochip

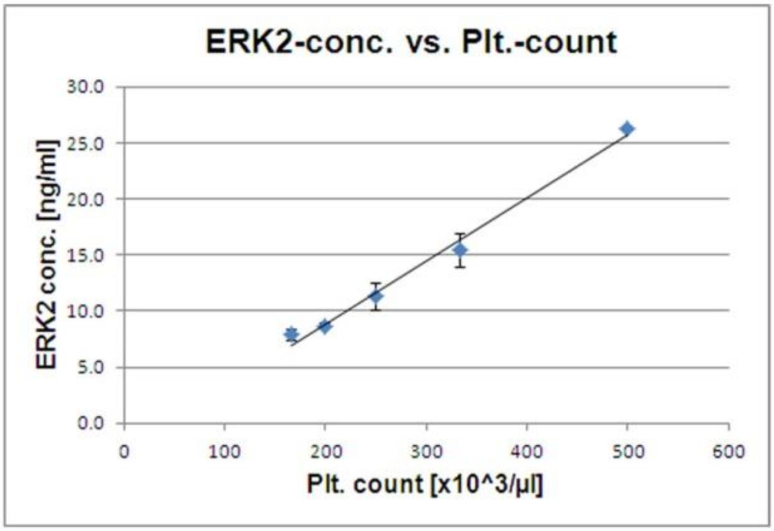

For normalization, ERK2 was intended to be compared with the well-established loading control GAPDH. Despite testing a variety of different anti-GAPDH antibodies, no sandwich assay could be established that fulfilled the strict requirements for normalization. To test the feasibility of ERK2, frozen PRP with a high platelet number of 509 x 10^3 platelets/µl was diluted four times (1/1.25, 1/1.5, 1/2.0, and 1/2.5) with autologous plasma. ERK2 concentrations of undiluted PRP and the four dilutions were quantified with the protein biochip in duplicates. Platelet numbers are stated on the x-axis, ERK2 concentrations on the y-axis.

Fig. OR6 Nine-point calibration curves for all protein biochip assays

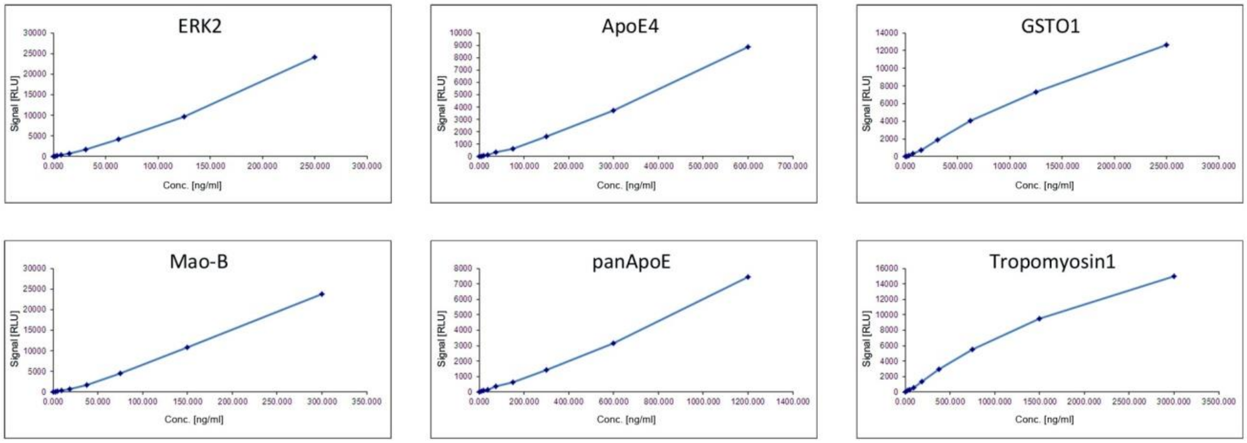

Concentrations of the recombinant calibrator proteins are indicated in ng/ml on the x-axis, relative light units (RLU) on the y-axis.

Tables

Table OR1 Demographic data of all study subjects

Demographic Variable	AD (*n* =62)	AD Co (*n* =63)	aMCI (*n* =24)	aMCI Co (*n* =24)	VaD (*n* =13)	VaD Co (*n* =13)	PD (*n* =12)	PD Co (*n* =12)
Mean age (±SD), [years]	82 (±7.2)	81 (±7.2)	85 (±5.5)	84 (±6.4)	81 (±9.1)	81 (±7.3)	65 (±7.6)	67 (±8.5)
MMSE (SD)	11 (±5.1)	29 (±1.2)	27 (±1.3)	28 (±1.2)	11 (±2.9)	28 (±1.1)	28 (±1.7)	28 (±1.5)
Female [%]	81	83	84	84	85	85	14	14
APOE ε4 $^{+/a}$ [%]	68	11	25	8	23	8	17	17
APOE ε4 $^{+/+}$ [%]	21	0	0	0	0	0	0	0
Platelet [c] *10^3/µl (±SD)	246 (±82)	251 (±67)	228 (±56)	199 (±75)	213 (±67)	240 (±72)	233 (±68)	214 (±73)
Education (±SD), [years]	11 (±3.6)	12 (±3.1)	12 (±3.0)	12 (±2.6)	n.a.	12 (±2.8)	n.a.	n.a.

[a]Percentage of *APOE ε4*-positivity (homo- or heterozygous). Co, control.

Table OR2 AD-related changes in the 2D-DIGE platelet proteome

Biomarker Candidates *n* = 10			Discovery *n* (AD) = 22 *n* (Co) = 25			Verification *n* (AD) = 40 *n* (Co) = 38			All *n* (AD) = 62 *n* (Co) = 63						AD Subgroups: Confirmed AD *n* = 9		AD Subgroups: *APOE ε4*-neg AD *n* = 20			AD Subgroups: *APOE ε4*-pos AD *n* = 42		
**Spot ID (890 spots analyzed)**	**Accession No.**	**Protein ID**	**Ratio (AD / Co)**	**Unadjusted *p*-value**	**Adjusted *p*-value**	**Ratio (AD / Co)**	**Unadjusted *p*-value**	**Adjusted *p*-value**	**Ratio (AD / Co)**	**Unadjusted *p*-value**	**Adjusted *p*-value**	**AUC**	**95% CI**	**Effect Size (ES)**	**Ratio (AD / Co)**	**Unadjusted *p*-value**	**Ratio (AD / Co)**	**Unadjusted *p*-value**	**Adjusted *p*-value**	**Ratio (AD / Co)**	**Unadjusted *p*-value**	**Adjusted *p*-value**
**B645**	**P27338**	**Monoamine oxidase B**	**1.28**	**< 0.001**	**0.029**	**1.46**	**< 0.001**	**< 0.001**	**1.38**	**< 0.001**	**< 0.001**	**0.823**	**0.749-0.898**	**1.50**	**1.31**	**0.003**	**1.38**	**< 0.001**	**0.021**	**1.38**	**< 0.001**	**< 0.001**
**A1942**	**P02649**	**Apolipoprotein E ε3**	**0.57**	**< 0.001**	**0.061**	**0.54**	**0.001**	**0.006**	**0.55**	**< 0.001**	**0.001**	**0.759**	**0.670-0.848**	**0.73**	**0.46**	**0.001**	**0.72**	**0.080**	**0.697**	**0.47**	**< 0.001**	**< 0.001**
**A1855**	**P09493**	**Tropomyosin 1**	**1.45**	**0.001**	**0.201**	**1.21**	**0.007**	**0.021**	**1.29**	**< 0.001**	**0.010**	**0.715**	**0.623-0.806**	**0.74**	**1.54**	**0.001**	**1.33**	**0.003**	**0.330**	**1.27**	**< 0.001**	**0.036**
**A1929**	**P02649**	**Apolipoprotein E ε4**	**3.31**	**0.001**	**0.201**	**1.88**	**0.006**	**0.021**	**2.22**	**< 0.001**	**0.001**	**0.797**	**0.694-0.899**	**1.18**	**1.96**	**0.020**				**2.53**	**< 0.001**	**< 0.001**
A921	P00488	Coagulation factor XIII A chain	1.27	0.003	0.334	1.15	0.245	0.420	1.19	0.007	0.296				1.30	0.030	1.23	0.051	0.662	1.14	0.037	0.425
B389	P51659	Peroxisomal multifunctional enzyme type 2	1.46	0.007	0.403	1.07	0.741	0.889	1.21	0.064	0.493				1.54	0.114	1.27	0.107	0.698	1.18	0.151	0.623
A916	P00488	Coagulation factor XIII A chain	1.26	0.007	0.403	0.87	0.977	0.977	1.02	0.117	0.577				1.11	0.681	0.93	0.769	0.948	1.03	0.107	0.430
A2006	P78417	Glutathione S-transferase omega1 mut = GSTO1*D140	0.72	0.031	0.679	0.88	0.123	0.246	0.80	0.009	0.316				0.80	0.315	0.43	< 0.001	0.095	0.93	0.419	0.790
A1663	P60709	Actin, cytoplasmic 1	1.35	0.039	0.679	0.83	0.543	0.815	1.01	0.543	0.815				1.12	0.662	1.01	0.237	0.795	0.92	0.791	0.949
A2000	P78417	Glutathione S-transferase omega1 mut = GSTO1*D140	0.70	0.039	0.679	0.95	0.623	0.831	0.84	0.095	0.547				0.70	0.351	0.41	< 0.001	0.037	0.98	0.972	0.995
A1998	***P78417***	***Glutathione S-transferase omega1 wt = GSTO1*A140***															**1.61**	**< 0.001**	0.020	**1.08**	**0.318**	**0.714**
**Further Isoforms of Tropomyosin which were indistinguishable by MS from A1855**																						
A1827	P09493	Tropomyosin 1							1.12	0.009	0.316				1.36	0.005						
A1896	P09493	Tropomyosin 1							1.07	0.080	0.528				1.17	0.282						
A1941	P09493	Tropomyosin 1							1.04	0.233	0.680				1.27	0.051						
SUM	P09493	Tropomyosin 1							1.13	0.018	0.382				1.29	0.034						
**Low biological variation protein as loading control for the platelet protein biochip**																						
B1115	P28482	Extracellular signal-regulated kinase 2							1.02	0.163	0.598											

Complete data set of Table 2. AD-specific protein spots are presented together with their AD/Co SA ratios, their unadjusted and adjusted *p*-values, and 95%CI. Date from the different study phases and the whole collective with *APOE*-stratified AD patients are shown. (Bold) proteins passed the biomarker verification and were selected for the protein biochip with ERK2 as loading control. Significances of *p*-values were calculated with the Mann-Whitney *U* test.

Table OR3 Performance of model 5 algorithm (Table 3) for identification of AD patients

Comparisons	AUC	95%CI
AD vs AD Co	0.969	0.944-0.994
AD vs. PD	0.912	0.824-1.000
PD vs PD Co	0.681	0.627-0.977
AD vs. VaD	0.738	0.540-0.936
VaD vs. VaD Co	0.802	0.682-0.876
aMCI vs. aMCI Co	0.798	0.700-0.897

Receiver operating characteristic (ROC) blots were computed from 2D-DIGE MaoB, Tm1, and GSTO1 SA combined with *APOE ε4* allele count. Resulting areas under the curve (AUC) were calculated for AD patients (*n*=62) vs. matched controls (AD Co, *n*=63), as well as for AD vs. Parkinson's disease (PD, *n*=12) and vascular dementia (VaD, *n*=13) patients. Furthermore, AUC were calculated for PD and VaD patients vs. matched cognitively healthy controls. To evaluate the diagnostic performance of this biomarker panel for a prodromal phase of AD, the AUC was also calculated for amnestic mild cognitive impairment (aMCI, *n*=24) patients vs. matched controls (*n*=24).

Text OR1 Biomarker validation with antibodies on 2D-WB

Thirty µg of trichloroacetic acid-precipitated and sample buffer-resolubilised platelet proteins were separated in the first dimension by IEF on a 13 cm pH 4-7 IPG strip. Second dimension SDS-PAGE was performed with 13 x 16 cm gels and resolved proteins were transferred onto nitrocellulose membranes (Pall, East Hills, NY, USA) which were blocked (2 h) with 5% (w/v) non-fat dry milk/PBS containing 0.3% Tween-20 (PBS-T). Proteins of interest were identified following incubation (1.5 h) with Randox sourced [monoclonal sheep anti-Tm1 exon 1a and exon 9d antibodies (both 1/1000), monoclonal anti-MaoB antibody ESC6449 (1/250), monoclonal anti-human ApoE4 antibody (1/2000)], and monoclonal goat anti-human panApoE antibody (1/2500; Innogenetics, Gent, Belgium). Primary antibody-specific binding was tagged with horseradish peroxidase-conjugated secondary antibodies (1/20,000 Jackson ImmunoResearch Laboratories, PA, USA) and visualized using a chemiluminescent substrate. The chemiluminescent signals were quantified with a Chemicon XRS system (Biorad, Hercules, CA, USA).

Antibodies

Since the identified proteins of interest displayed regulation at both the DNA and the protein level, most of the targets were specific isoforms with no commercial antibodies available. Thus, antibody generation was completed in-house. Tropomyosin was a particularly challenging target due to the variety of isoforms generated by alternative splicing, leading to different exon compositions [3]. Three spots (A1827, A1896, and A1941; Fig. 1) in the vicinity of the AD-regulated Tm1 spot A1855 were also identified as Tm1 but could not be differentiated further by MS. These spots were either unchanged or just slightly up-regulated in AD (Fig. OR1c). Since the combination of A1855-specific exons 1a and 9d was expected to help differentiating several Tm1 isoforms, monoclonal antibodies were raised by immunization of sheep with synthesized

and conjugated 21 and 27 residue peptides. As depicted in Fig. OR1a, the antibody directed against exon 9d recognized proteins of the spots A1855, A1896, and A1941, but only weakly A1827. Additionally, it bound to the proteins of spots A2018 (Tm1, P09493-2) and A1987 (Tm4, P67936), both unchanged in AD patients. The exon 1a-specific antibody, however, bound just high molecular weight Tm1 isoforms (A1827, A1855, A1896, and A1941). Thus, Tm1 spots A1855, A1896, and A1941 could be assigned to P09493-3, A1827 to P09493-1, and spots A2018 to P09493-2. An antibody sandwich pair consisting of one of the specific antibodies as capture and one as detector should pick up the three P09493-3 isoforms, containing the strongly regulated A1855 and the weaker regulated A1896 and A1941 spots with high specificity. The 2D-DIGE sum score of these three Tm1-3 spots was significantly increased by 13% (p=0.028) in AD patients. Remarkably, the biochip tropomyosin concentrations correlated best with the SA of spot A1855 (r=0.68), the strongest AD-regulated Tm1 isoform, as compared to A1827, A1896, and A1941.

GSTO1*D140 and ApoE4 protein isoforms resulted from SNPs, thus two anti-GSTO1 monoclonal antibodies, one directed against GSTO1*A140 and one against panGSTO1 were developed. Their specificity has been reported in our last publication (as well as that of the anti-ERK2 antibody) and allowed proteomic genotyping in a gel-based system [5]. Similarly, to distinguish the ApoE isoform E4 from E2 and E3, which differ in amino acids at positions 112 and 158, a monoclonal antibody specific for the E4 isoform was generated. The blot (Fig. OR1a) confirmed no crossreactivity of the antibody with E2 or E3 proteins. Consistently, only the anti-panApoE antibody recognized the ApoE3 spot in an *APOE ε3/ε3* platelet sample. As indicated in Fig. OR1b, also the anti-MaoB monoclonal antibody was highly specific: apart from the dominant MaoB spot, the minor spot recognized by the antibody could also be identified as MaoB.

Text OR2 Protein biochip analysis

Capture antibodies were automatically spotted onto the biochip surface at Randox Laboratories: anti-ApoE4, anti-MaoB, anti-Tm1 exon 9d, anti-panGSTO1 (Randox Laboratories), anti-ERK2 (Santa Cruz, Santa Cruz, USA), anti-panApoE (Innogenetics, Gent, Belgium). Biochips were chopped, sealed, and stored at 4 ˚C. For quantification, 100 µl sample lysate or calibrator was added to 200 µl assay buffer (Randox Laboratories) provided in the biochip wells and biochips were incubated (37 ˚C, 1 h) under gentle agitation (370 rpm). Biochips were washed six times (2x quick, 4x 2 min) with wash buffer containing 0.5% Tween and incubated with 300 µl target-specific HRP-labeled antibodies (37 ˚C, 1 h). After washing (2x quick, 4x 2 min), 250 µl signal reagent (Luminol PLUS/Peroxide, Randox Laboratories) was added and biochips were incubated for 2 min in the dark before recording of the chemiluminescent signal using the 'Evidence Investigator' analyzer (Randox Laboratories). A grid was superimposed onto the acquired images and aligned with the help of two reference spots for correct spot detection. Signal intensities were quantified with the 'Randox Imager' software and subsequently processed with standard Microsoft Excel software.

OR References

1. Baumgartner R, Umlauf E, Veitinger M, Guterres S, Rappold E, Babeluk R, Mitulovic G, Oehler R, and Zellner M (2013) Identification and validation of platelet low biological variation proteins, superior to GAPDH, actin and tubulin, as tools in clinical proteomics. J Proteomics 94C:540-551.

2. Burkhart JM, Vaudel M, Gambaryan S, Radau S, Walter U, Martens L, Geiger J, Sickmann A, and Zahedi RP (2012) The first comprehensive and quantitative analysis of human platelet protein composition allows the comparative analysis of structural and functional pathways. Blood 120:e73-e82.

3. Schevzov G, Vrhovski B, Bryce NS, Elmir S, Qiu MR, O'neill GM, Yang N, Verrills NM, Kavallaris M, and Gunning PW (2005) Tissue-specific tropomyosin isoform composition. J Histochem Cytochem 53:557-570.

4. Slany A, Haudek VJ, Gundacker NC, Griss J, Mohr T, Wimmer H, Eisenbauer M, Elbling L, and Gerner C (2009) Introducing a new parameter for quality control of proteome profiles: consideration of commonly expressed proteins. Electrophoresis 30:1306-1328.

5. Veitinger M, Umlauf E, Baumgartner R, Badrnya S, Porter J, Lamont J, Gerner C, Gruber CW, Oehler R, and Zellner M (2012) A combined proteomic and genetic analysis of the highly variable platelet proteome: from plasmatic proteins and SNPs. J Proteomics 75:5848-5860.

6. Winkler W, Zellner M, Diestinger M, Babeluk R, Marchetti M, Goll A, Zehetmayer S, Bauer P, Rappold E, Miller I, Roth E, Allmaier G, and Oehler R (2008) Biological variation of the platelet proteome in the elderly population and its implication for biomarker research. Mol Cell Proteomics 7:193-203.

7. Zellner M, Baureder M, Rappold E, Bugert P, Kotzailias N, Babeluk R, Baumgartner R, Attems J, Gerner C, Jellinger K, Roth E, Oehler R, and Umlauf E (2012) Comparative platelet proteome analysis reveals an increase of monoamine oxidase-B protein expression in Alzheimer's disease but not in non-demented Parkinson's disease patients. J Proteomics 75:2080-2092.

8. Zellner M, Winkler W, Hayden H, Diestinger M, Eliasen M, Gesslbauer B, Miller I, Chang M, Kungl A, Roth E, and Oehler R (2005) Quantitative validation of different protein precipitation methods in proteome analysis of blood platelets. Electrophoresis 26:2481-2489.
